# Exposure to Forced Swim Stress Alters Local Circuit Activity and Plasticity in the Dentate Gyrus of the Hippocampus

**DOI:** 10.1155/2008/194097

**Published:** 2008-02-14

**Authors:** Orli Yarom, Mouna Maroun, Gal Richter-Levin

**Affiliations:** ^1^Department of Psychobiology, Faculty of Sciences, University of Haifa, Haifa 31905, Israel; ^2^The Brain and Behavior Research Center, University of Haifa, Haifa 31905, Israel

## Abstract

Studies have shown that, depending on its severity and context, stress can affect neural plasticity. Most related studies focused on synaptic plasticity and long-term potentiation (LTP) of principle cells. However, evidence suggests that following high-frequency stimulation, which induces LTP in principal cells, modifications also take place at the level of complex interactions with interneurons within the dentate gyrus, that is, at the local circuit level. So far, the possible effects of stress on local circuit activity and plasticity were not studied. Therefore, we set out to examine the possible alterations in local circuit activity and plasticity following exposure to stress. Local circuit activity and plasticity were measured by using frequency dependant inhibition (FDI) and commissural modulation protocols following exposure to a 15 minute-forced swim trial. Exposure to stress did not alter FDI. The application of theta-burst stimulation (TBS) reduced FDI in both control and stressed rats, but this type of plasticity was greater in stressed rats. Commissural-induced inhibition was significantly higher in stressed rats both before and after applying theta-burst stimulation. These findings indicate that the exposure to acute stress affects aspects of local circuit activity and plasticity in the dentate gyrus. It is possible that these alterations underlie some of the behavioral consequences of the stress experience.

## 1. INTRODUCTION

Stress is defined as any condition that seriously disrupts physiological and psychological homeostasis ranging from anxiety to posttraumatic stress disorder [1], and affects cognitive functions both in animal models and in humans [2–4]. The hippocampus is of special significance in this respect because it has been shown to play a major role in regulating stress [5, 6], and to be involved in some aspects of learning and memory [7–13].

At present, long-term potentiation (LTP) of synaptic transmission in the hippocampus is the most studied neurophysiological model for learning and memory processes in the mammalian nervous system. LTP, like behavior, appears to be affected by stress. Depending on the type of stress and the procedures used, stress has been shown to have different effects on different measures of synaptic plasticity. There is a general agreement that LTP in area CA1 of the hippocampus is impaired following stress [4, 14–18]. Some studies have also shown that stress impairs LTP in the dentate gyrus (DG) of the hippocampus [16, 19, 20], while others reported intact LTP in the DG following stress [14, 21]. Thus, DG LTP is considered to be less sensitive to stress compared to LTP in CA1 [22].

Although LTP is a widely accepted model of learning and memory, debates continue over its validity, and controversial results regarding its behavioral correlates are reported (for review, see [23]). A different level of processing that is likely to be relevant to memory formation is local circuit activity. When examining this level of processing, the focus is on interactions between local, mostly inhibitory GABAergic neurons and pyramidal or granular principle cells in the hippocampus and cortex [24, 25]. This is in contrast to the focus on LTP of input excitatory synapses onto principle cells, which is responsible for transmitting information from one region to another. Inhibitory interneurons exert a powerful control over local circuit activity through feedforward and feedback inhibition. Modification of local circuits can affect the computational properties of the region, and therefore affect its involvement in behavior.

In the current study, local circuit activity and plasticity were measured by using frequency-dependent inhibition (FDI) and commissural modulation protocols, following exposure to behavioral stress.

FDI is suggested to reflect GABA-mediated inhibition by perforant path- (PP-) activated interneurons onto granule cells [26]. Increasing stimulus frequency from 0.1 Hz to 1.0 Hz results in the reduction of the population spike (PS) of the field potential response to stimulation of the PP [27]. Our lab has previously shown that FDI in the DG is NMDA-dependent [28], GABA-mediated, and that delivering theta-burst stimulation (TBS) to the PP of the hippocampus induced a lasting reduction in FDI [18].

The DG commissural pathway is activated by stimulating the contralateral DG at different intervals prior to PP stimulation. Stimulation of the commissural pathway induces a biphasic, inhibitory/excitatory effect on granule cell responsiveness to PP stimulation. The inhibitory phase is a result of activation of feedforward inhibition [29].

Although the effect of behavioral stress on induction of hippocampal LTP has been studied extensively, to our knowledge no research has established the relationship between stress and local circuit activity and plasticity. The current study addresses this issue in order to further explore the potential relevance of local circuit activity to learning and memory. Our aim in this study was to characterize local circuit activity and plasticity in the DG of the hippocampus following exposure to behavioral stress.

## 2. METHODS

### 2.1. Subjects

Adult, male Sprague Dawley rats, weighing 240–330 g, from Harlan (Jerusalem, Israel) maintained five per cage on a 12-hour light/dark cycle with water and laboratory rodent chow *ad libitum*.

### 2.2. Corticosterone radioimmunoassay

Trunk blood was collected following decapitation and samples were centrifuged at 3000 r.m.p. for 20 minutes at 4°C. Serum was stored at −80°C. Corticosterone was measured using a radioimmunoassay kit (Coat-A-Count, Diagnostic Products Corporation, Los Angeles, Calif, USA).

### 2.3. Electrophysiology

Rats were anaesthetized (6% chloral hydrate in 100 mL saline; 0.5 mL/100 g. IP) and prepared for acute stimulation of the perforant path and for recording of field potentials in the dentate gyrus as described before [29].

Rats were placed in a head holder in a stereotaxic frame and small holes were drilled in the skull to allow the insertion of electrodes in the brain. A recording microelectrode (glass, tip diameter 2–5 *μ*m filled with 2 M NaCl, resistance 1–4 M*Ω*) was placed in the dentate gyrus (coordinates: 4 mm posterior to bregma, 2.5 mm lateral to midline). A bipolar 125 *μ*m stimulating electrode was implanted in the ipsilateral angular bundle to stimulate the perforant path (coordinates: 8 mm posterior to bregma, 4 mm lateral to midline). The depth of the electrodes was adjusted to maximize the size of the evoked positive-going excitatory postsynaptic potential (EPSP) recorded in the hilus of the dentate gyrus.

Evoked responses were digitized (10 kHz) and analyzed using the Cambridge Electronic Design 1401+ and its Spike2 software. Offline measurements were made of the amplitude of the PS and the slope of the EPSP using averages of 5 successive responses to a given stimulation intensity applied at 0.1 Hz. Test stimuli (monopolar pulses, 100-microsecond duration, intensity adjusted to yield a PS of 30–50% of the maximal pretetanus value) were delivered at 0.1 Hz. After positioning the electrodes, the rat was left for 20 minutes before commencing the experiment. During recording the rats were maintained at 37 ± 1°C with a homeothermic blanket system (Harvard).

### 2.4. Long-term potentiation

LTP was induced by a TBS (3 sets of 10 trains, each consisting of 10 pulses at 100 Hz. Intertrain interval: 200 milliseconds, and the interval between each set: 1 minute, trains are delivered at 2x test stimulus intensity).

LTP was measured as the difference in EPSP slope before and 60 minutes after TBS. We defined LTP as an increase of the least 20% in the EPSP slope of the evoked potentials 60 minutes after application of TBS.

### 2.5. Local circuit activity

**Frequency-dependent inhibition**

To determine FDI, 10 pulses were delivered at 0.1 Hz followed by 10 pulses at 1.0 Hz, as described before [18]. This pattern was repeated twice. The pulses given were at test stimulus intensity. The PS or EPSP slope of the 10 responses at 0.1 Hz were averaged and compared to the 10 responses at 1.0 Hz in each set. The results of the two sets were averaged. Inhibition is expressed as an FDI index which was assessed by dividing the averaged response at 1.0 Hz by the averaged response at 0.1 Hz.

**Commissural-induced modulation**

The DG commissural pathway was activated by stimulating the contralateral DG at different intervals prior to PP stimulation (15, 30, 80, and 150 milliseconds) as described before [30]. Stimulation of the commissural pathway induces a biphasic inhibitory/excitatory effect on granule cell responsiveness to PP stimulation [31, 32].

Commissural-induced modulation is expressed by commissural index which was evaluated as the ratio of the size of the response to PP stimulation after commissural stimulation divided by that of the response to PP stimulation with no priming stimulation.

In order to measure TBS-induced alterations on frequency-dependent inhibition or on commissural-induced inhibition and facilitation, the stimulation intensity following the induction of LTP was adjusted to yield a PS size comparable to pre-TBS level.

### 2.6. Behavior

#### 2.6.1. Elevated plus maze

**Apparatus**

The maze employed is a four-armed black opaque Plexiglas platform, elevated 50 cm above ground. Two opposite arms are enclosed by 40 cm high Plexiglas walls on both sides and on the outer edges of the platform, that is, “closed,” while the two remaining opposite arms are “open,” and are surrounded only by a 1 cm high Plexiglas rim, which serves as a tactile guide to animals in the open areas. Individual rats were placed in the central platform, faced towards different arms in randomized order.

**Procedure**

Each five-minute session was recorded using an overhead video camera connected to a monitor/recorder in an adjacent observation room. Animals were placed in the central platform and allowed to explore for five minutes. Animals were then subjected to the acute swim stress procedure, and then allowed to rest for 90 minutes. After the resting period animals were placed once again in the maze for a five-minute poststress test. Time spent in the open arms was measured. Animals were scored as being in an open or closed arm only when all four paws passed over the open/closed dividing line.

### 2.7. Induction of behavioral stress

**Apparatus**

The water container used for the forced swim procedure consists of a circular container of water (50 cm in diameter with a rim 75 cm high). Water depth was 50 cm and temperature was maintained at 23 ± 1°C.

**Acute Swim Stress (ASS) procedure**

Rats were subjected to ASS as previously described [33]. Individual rats were subjected to a single 15-minute swim session in the water container. After this single swim session, rats were allowed to dry in a resting cage for 30 minutes, and then anesthetized and taken to electrophysiology.

## 3. RESULTS

### 3.1. Acute swim stress induces elevated levels of serum corticosterone and increased levels of anxiety

**Elevated plus maze**

In order to validate that animals subjected to the acute swim stress procedure were indeed behaviorally affected, we used the elevated plus maze test [34].

**Figure 1:**
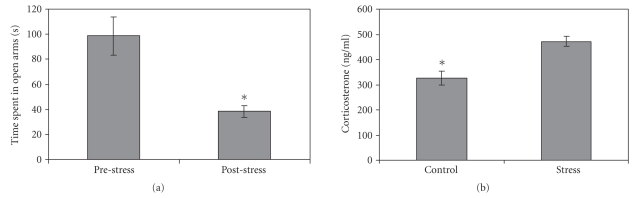
(a) *Time spent in open arms of the elevated plus maze before and after exposure to stress*. Following exposure to acute swim stress, rats spent less time in the open arms of the maze in comparison to the time spent in the open arms prior to the stress procedure (*n* = 8, *P* < .005), indicating increased anxiety. (b) *Levels of corticosterone in control and stress rats*. Acute swim stress induced elevation of serum corticosterone in the stressed rats (*n* = 6) compared to control rats (*n* = 8, *P* < .005).

A paired sample *t*-test revealed that in the poststress session, animals spent less time in the open arms (see Figure 1(a), t(7) = 4.25, *P* < .005).

**Levels of corticosterone**

Levels of serum corticosterone were measured for control and stressed rats. An independent *t*-test revealed that stress was associated with a significant increase in corticosterone levels (see Figure 1(b), t(13) = 4.29, *P* < .005) (see Figure 1(b)).

### 3.2. Acute swim stress does not affect baseline responses in PP-DG pathway

The stimulation intensity used to elicit a baseline response was not different between the control and stress groups (t(25) = 0.93, n.s.). There was no significant difference between control and stressed rats in the amplitude of the baseline PS (t(25) = 1.44, n.s.)) and fEPSP slope (t(25) = 0.26, n.s.).

### 3.3. The effect of behavioral stress on frequency-dependent inhibition

**Figure 2:**
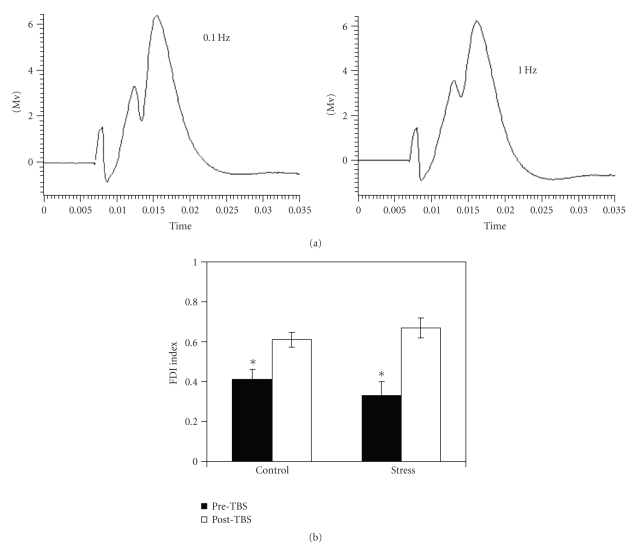
(a) Left: representative field potential response of dentate gyrus granule cells to stimulating of the PP at 0.1 Hz. Right: representative field potential response of dentate gyrus granule cells to stimulation of the PP at 1.0 Hz. Time unit: second. (b) *FDI before and after TBS application*. The application of TBS significantly reduced FDI in both control (*n* = 8) and stressed (*n* = 8) rats (*P* < .05).

Upon altering the frequency of stimulation from 0.1 Hz to 1.0 Hz, a marked reduction of the PS was observed in both control and stressed rats as indicated by the FDI index (see Figure 2(a)). This reduction was apparent prior to TBS application and 60 following it (see Figure 2(b), F(1,14) = 101.16, *P* < .05). No significant differences in FDI index were found between the two groups on both tests (F(1,14) = 0.18, n.s.)

Although no main effect was observed for group under the different conditions, the analysis of variance indicated a significant group X test interaction (F(1,14) = 6.53, *P* < .05). The initial pre-TBS FDI index of stressed rats was lower than that of controls whereas the post-TBS FDI index was higher.

Altering stimulation frequency from 0.1 Hz to 1.0 Hz resulted in a slight but significant reduction in the EPSP slope in both groups. In control rats, stimulation at 1.0 Hz reduced the slope of the EPSP to 0.85±0.05 of its size during stimulation at 0.1 Hz (t(7) = 11.49, *P* < . 05). In stressed rats, stimulation at 1.0 Hz reduced the slope of the EPSP to 0.88±0.02 of its size during stimulation at 0.1 Hz [t(7) = 3.66, *P* < 0.05]. There was no significant difference in EPSP FDI between the two groups and, in contrast to the PS, the application of TBS did not affect EPSP FDI in either group.

### 3.4. The effects of behavioral stress on commissural modulation

**Figure 3:**
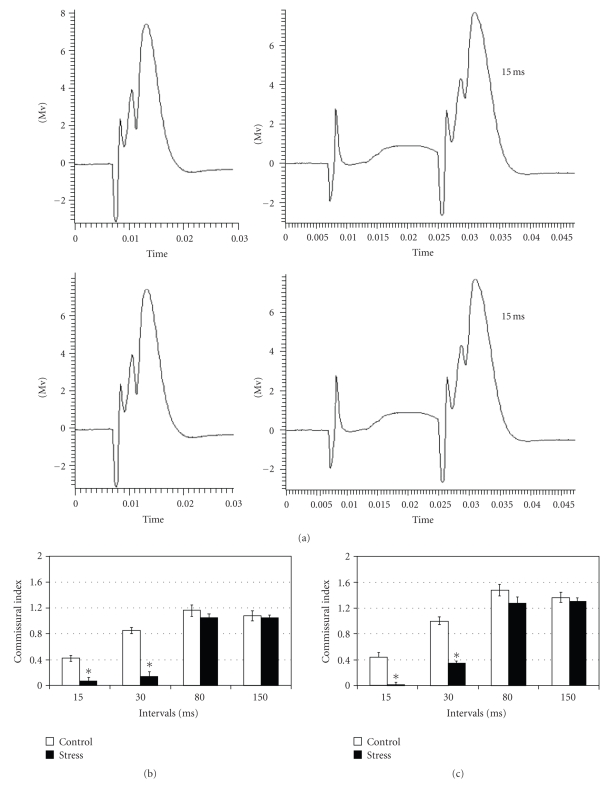
(a) *The effects of commissural priming on responses of the DG to PP stimulation*. Top: representative field potential responses of dentate gyrus cells to stimulating the PP without commissural priming (left), and to stimulating the PP with priming stimulation to the contralateral DG at 15 milliseconds (right) in control rats. Bottom: representative field potential responses of dentate gyrus cells to stimulating the PP without commissural priming (left), and to stimulating the PP with priming stimulation to the contralateral DG at 15 milliseconds (right) in stressed rats. Time unit: second. (b) *Commissural-induced modulation prior to TBS application*. Commissural-induced inhibition was significantly higher in stressed rats (*n* = 6) at 15-millisecond and 30-millsecond interpulse intervals in comparison with control rats (*n* = 6) (*P* < .05). (c) *Commissural-induced modulation following TBS application*. Commissural-induced inhibition was significantly higher in stressed animals at 15-millisecond and 30-millisecond intervals *n* = 6 (*P* < .05).

In both control and stressed rats, the response to the PP stimulation was inhibited by priming stimulation of the commissural path at interpulse intervals of 15 milliseconds and 30 milliseconds (see Figure 3(a)). When using the 15-millisecond interpulse interval, exposure to stress induced significantly higher inhibition, as expressed by the small commissural index of stressed rats compared to controls. This increase in inhibition was apparent before and after TBS application (F(1,12) = 64.36, *P* < .005). The application of TBS did not cause any significant changes in commissural-induced inhibition in either group (F(1,12) = 0.126, n.s) (see Figures 3(b) and 3(c)).

When priming stimulation of the commissural path at interpulse interval of 30 milliseconds, stressed rats exhibited significantly higher commissural-induced inhibition than control rats before and after TBS application (F(1,12) = 90.42, *P* < .001), as seen in the 15-millisecond interpulse interval. Interestingly, both groups have shown a significant decrease in commissural-induced inhibition following TBS application (F(1,12) = 13.68, *P* < .005). (see Figures 3(b) and 3(c)).

When using the 80-millisecond and 150-millisecond interpulse intervals, the response to the PP stimulation was facilitated in both control and stressed rats, as expressed by the large commissural indexes. In both groups, commissural-induced facilitation was significantly increased after TBS application (80 milliseconds: F(1,12) = 17.55, *P* < .005; 150 milliseconds: F(1,12) = 7.87, *P* < .05). No differences were found between the two groups before or after TBS application (80 milliseconds: F(1,12) = 0.68, n.s; 150 milliseconds: F(1,12) = 0.057, n.s). (See Figures 3(b) and 3(c)).

### 3.5. The effect of the stressor on LTP induction in the dentate gyrus

Sixty minutes following the application of TBS, there was a clear potentiation of the slope of the EPSP in control rats (38% ± 5.9, t(13) = 6.38, *P* < .001) and stressed rats (35% ± 9.3, t(10) = 3.54, *P* < .05), compared to pre-TBS levels. The application of TBS also resulted in an increase in PS amplitude in both groups (control: 128% ± 32, t(13) = 4.02, *P* < .005); stress: 127% ± 38, t(10) = 4.6, *P* < .05).

**Figure 4:**
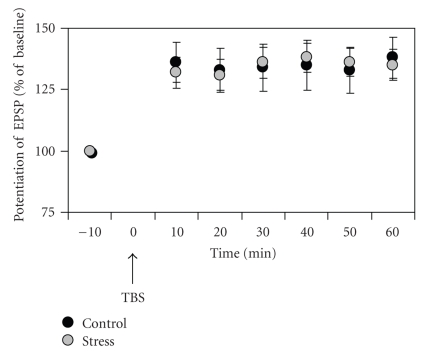
*TBS-induced potentiation in control and stress rats*. Both control (*n* = 14) and stress (*n* = 11) rats have shown an increase in the slope of the EPSP. The magnitude of the EPSP potentiation was not significantly different between the two groups.

No significant difference in LTP was found between the two groups (EPSP-LTP: t(23) = 0.33; PS-LTP: t(23) = 0.93, n.s)] (see Figure 4).

## 4. DISCUSSION

In the present study, we have examined the effects of behavioral stress on local circuit activity and plasticity in the DG. We report that when using FDI [26], this form of local circuit activity was reduced following the application of TBS in both control and stressed rats. However, this reduction in FDI plasticity was greater in the stressed rats compared to controls.

When using commissural-induced modulation, inhibition was significantly higher in stressed rats both before and after TBS at 15- and 30-millisecond intervals.

FDI results from direct afferent excitation of inhibitory interneurons or of other cells that excite inhibitory cells [26, 35], and thus provides a simple method for measuring local circuit activity mediated by dentate interneurons. In the present study, an attempt was made to find out whether behavioral stress would have an effect on FDI activity and plasticity. Our results reveal that both control and stressed rats have shown a decrease in FDI following TBS, and that differences between pre- and posttetanic levels of FDI were not significantly different between the two groups. When examining the differences between pre- and post-TBS levels of FDI within each group, a greater difference was observed in the stressed group, which may suggest that undergoing behavioral stress has caused an increase in FDI plasticity.

This increased plasticity is somewhat surprising since exposure to stress is typically shown to suppress plasticity and to impair learning [36–40]. It should be noted though that most studies related to stress effects on plasticity focused mainly on LTP and on CA1 area of the hippocampus. The effects of stress on LTP in other brain regions are less consistent [14, 21, 41]. Furthermore, even in CA1, while stress may suppress LTP, it is reported to enhance other forms of plasticity, such as LTD [17, 42].

The effects of stress on commissural-induced modulation were also examined. Stressed rats have shown a marked increase in inhibition both before and after the application of TBS, indicating a lasting modification in this form of local circuit activity. This result is in agreement with other studies that have shown that stress levels of corticosterone produce a profound and long-lasting inhibitory influence on hippocampal cell activity [43–47]. Although stress has caused a reduction in local circuit activity in this case, no alteration in commissural local circuit plasticity was observed after TBS application.

The fact that FDI activity was not affected by the stress while commissural-induced modulation was significantly affected suggests that stress may affect only a subpopulation of inhibitory interneurons. Indeed, it has been previously suggested that interneurons in the hippocampus may be divided into those showing no activity-dependent plasticity and those that do. It was further suggested that activity-dependnet plasticity of this sort may contribute to mood and anxiety disorders [48].

The fact that an increase in inhibitory activity was observed in the hippocampus may imply that deficits in cognitive functioning and flexibility might take place, as has been suggested before for aged rats [18]. This does not necessarily mean that rats that have undergone stress would show impaired learning in a specific task, but it is possible that they would be less adaptive if required to shift to a new coping strategy during task acquisition. For example, stress has been shown to cause impairment in reversal learning and induced perseveratory behavior in the Morris water maze without having a significant effect on task acquisition [49].

In the present study, dentate gyrus LTP was not affected by the stress employed. As indicated above, the effects of stress on dentate gyrus LTP may depend on the exact nature of the stress experience [14, 21, 41]. Interestingly, although stress seemed to have caused alterations in local circuit plasticity (as observed in the FDI findings) and in local circuit activity (as observed by the commissural modulation findings), it had no effect on LTP induction. This further supports the notion that local circuit activity and plasticity is independent of synaptic plasticity such as LTP [18, 28].

The study of the effects of stress on GABAergic neurotransmission is of special interest because it has been suggested that GABA plays a role in the pathophysiology of mood and anxiety disorders [50–54]. The results presented here may further support the potential role of GABA-impaired modulation of neural activity and plasticity in stress-related disorders.

Our results suggest that stressful experience may lead to alterations in local circuit activity and plasticity. Understanding the alterations that take place in these local interneurons may contribute to a better understanding of their involvement in memory formation and regulation under normal and psychopathological conditions.
